# Acceleration dependence and task-specific modulation of short- and medium-latency reflexes in the ankle extensors

**DOI:** 10.1002/phy2.51

**Published:** 2013-08-22

**Authors:** James M Finley, Yasin Y Dhaher, Eric J Perreault

**Affiliations:** 1 Department of Biomedical Engineering, Northwestern UniversityEvanston, Illinois, USA; 2 Sensory Motor Performance Program, Rehabilitation Institute of ChicagoChicago, Illinois, USA; 3 Department of Physical Medicine and Rehabilitation, Northwestern UniversityEvanston, Illinois, USA

**Keywords:** Acceleration, ankle, long-latency reflex, stability

## Abstract

Involuntary responses to muscle stretch are often composed of a short-latency reflex (SLR) and more variable responses at longer latencies such as the medium-latency (MLR) and long-latency stretch reflex (LLR). Although longer latency reflexes are enhanced in the upper limb during stabilization of external loads, it remains unknown if they have a similar role in the lower limb. This uncertainty results in part from the inconsistency with which longer latency reflexes have been observed in the lower limb. A review of the literature suggests that studies that only observe SLRs have used perturbations with large accelerations, possibly causing a synchronization of motoneuron refractory periods or an activation of force-dependent inhibition. We therefore hypothesized that the amplitude of longer latency reflexes would vary with perturbation acceleration. We further hypothesized that if longer latency reflexes were elicited, they would increase in amplitude during control of an unstable load, as has been observed in the upper limb. These hypotheses were tested at the ankle while subjects performed a torque or position control task. SLR and MLR reflex components were elicited by ankle flexion perturbations with a fixed peak velocity and variable acceleration. Both reflex components initially scaled with acceleration, however, while the SLR continued to increase at high accelerations, the MLR weakened. At accelerations that reliably elicited MLRs, both the SLR and MLR were reduced during control of the unstable load. These findings clarify the conditions required to elicit MLRs in the ankle extensors and provide additional evidence that rapid feedback pathways are downregulated when stability is compromised in the lower limb.

## Introduction

When posture is threatened by unexpected perturbations, stretch reflexes provide a rapid motor response to oppose the perturbation and restore stability. Accordingly, a number of studies in the human upper limb have demonstrated that the amplitude of the stretch reflex is preferentially enhanced during postural tasks requiring active stabilization of an external load (Doemges and Rack 1992a,b; Dietz et al. 1994; Perreault et al. 2008; Shemmell et al. 2009; Krutky et al. 2010). This modulation has been observed only in the long-latency stretch reflex (LLR), which occurs after approximately 50 msec in the human upper limb. In the lower limb, two distinct longer latency responses occur after the short-latency reflex (SLR): a medium-latency reflex (MLR) and an LLR (Toft et al. 1989, 1991). Although it has been demonstrated that SLRs can be modulated in the lower limb (Ludvig et al. 2007), we are unaware of any lower limb studies reporting load-dependent MLR or LLR modulation. This absence of information may result from the inconsistency with which these responses in the lower limb are observed in the literature. Hence, the objectives of this study were to identify conditions in which longer latency responses could be evoked in the human lower limb, and to examine how these responses are modulated in tasks that compromise limb stability.

Longer latency responses are only sometimes reported in studies examining lower limb stretch reflexes. Gottlieb and Agarwal (1979) described two reflex components in the ankle extensors, an SLR starting at approximately 45 msec and a more variable longer latency component at approximately 60–65 msec. The existence of two distinct reflex components has also been corroborated by a number of other studies (Fellows and Thilmann 1989; Toft et al. 1991; Fellows et al. 1993; Grey et al. 2002; Kimura et al. 2003). These responses were elicited using perturbations of various peak velocities (100–300°/sec) and amplitudes (5–10°), suggesting that the presence of long- and short-latency reflex components is insensitive to a range of perturbation characteristics. However, numerous other studies using comparable perturbation amplitudes and velocities report clear SLRs, but no discernible MLRs or LLRs (Allum et al. 1982; Kearney and Chan 1982; Nielsen et al. 1994; Avela et al. 1999; Finley et al. 2012). This discrepancy in the observation of longer latency responses makes it difficult to discern their functional role, and the range of lower limb tasks and perturbations for which they may be relevant.

Despite the wide range of perturbation parameters used in previous studies, those reporting longer latency responses often use a perturbation with a smooth or gradual onset. For example, the bell-shaped velocity profile presented in Fellows et al. (1993) is indicative of low acceleration and deceleration at the beginning and end of the ramp perturbation. In contrast, studies with the most rapid transitions always report only SLRs (Stein and Kearney 1995; Mirbagheri et al. 2000; Finley et al. 2012). Although accelerations are not often reported, it is possible that high initial accelerations may suppress longer latency reflex components by either synchronizing the refractory periods of the relevant motoneurons or increasing the input from inhibitory Ib afferents. The amplitude of LLRs elicited in the upper limb also depends on perturbation duration (Lee and Tatton 1982; Lewis et al. 2005). Therefore, if high accelerations are coupled with rapid velocities or small displacements, it is possible that perturbations will be of insufficient duration to evoke longer latency reflexes. Indeed, it has been suggested that the LLR in lower limb may be more prevalent following perturbations having low acceleration (Berardelli et al. 1982). However, this conclusion was reached by applying perturbations with a wide range of peak velocities (100–250°/sec) while simultaneously changing perturbation acceleration. Therefore, as acceleration was not independently varied, it remains unclear whether these observations reflected changes in acceleration or velocity.

The purpose of this study was to quantify the influence of perturbation acceleration on longer latency responses in the ankle extensors, and to examine if these responses, when present, are modulated during interaction with different mechanical environments that challenge ankle stability. The study involved two separate experiments. Our initial experiment tested the hypothesis that the amplitude of longer latency responses would vary nonlinearly with the initial acceleration of the imposed perturbation: increasing at low accelerations as stretch-sensitive afferents are recruited, reaching a maximum at modest accelerations, and finally attenuating as acceleration is further increased. Our second experiment then tested the hypothesis that the amplitude of longer latency responses would increase during interactions with mechanical environments that challenge ankle stability, consistent with the proposed stabilizing role for these responses. The outcome of this study may help to elucidate the stimulus characteristics that influence longer latency responses in the lower limb and further our knowledge of the stabilizing role of these reflex components during postural tasks.

## Materials and Methods

### Subjects

Thirteen subjects (7 males and 6 females, ages 25–33) who had no prior history of injury to the ankle participated in this study. Seven subjects participated in the first experiment and six subjects participated in the second experiment. All experimental procedures were submitted to, and approved by, the Institutional Review Board of Northwestern University (IRB protocol #0673-010) and complied with the principles of the Declaration of Helsinki. Written informed consent was obtained prior to testing.

### Equipment

**Figure 1:**
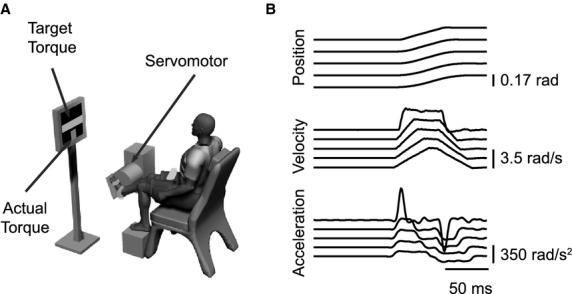
Experimental setup and perturbations used to examine the acceleration dependence of stretch reflexes in the ankle extensors. (A) Subjects were seated with the knee fixed at 0.52 rad (30°) of flexion and the ankle at 0.17 rad (10°) of ankle extension. (B) Position, velocity, and acceleration for each perturbation are shown from top to bottom. For all perturbations, displacement and peak velocity were fixed at 0.17 rad and 3.5 rad/sec (200°/sec), respectively. Acceleration was varied from a minimum of 87 rad/sec^2^ (bottom trace) to a maximum of 873 rad/sec^2^ (top trace). This variation resulted in stretch durations ranging from 50 to 90 msec.

A computer-controlled brushless servomotor (BSM90N-3150AX; Baldor Electric Company, Fort Smith, AR) was used to apply angular rotations to the ankle joint. Angular feedback was provided by an encoder with an effective resolution of 6.3 × 10^−5^ rad. Forces and moments were measured using a six-degree-of-freedom load cell (630N80; JR3, Inc, Woodland, CA) and the motor was controlled in real-time using Matlab xPC®. The servomotor system had a bandwidth of 125 Hz. A number of safety mechanisms were implemented in the system including: software-based amplitude limits of ±15°, inductive proximity sensors (TL-W5E; Omron Industrial Automation, Schaumburg, IL), an emergency stop button to cut power to the motor, and hard stops at the end of the movement range. Subjects were seated in a Biodex chair and movement of the trunk was minimized using a set of straps placed across the torso. Each subject's right knee was flexed 0.52 rad (30°) and the foot was secured to an aluminum footplate using straps placed across the forefoot and heel (Fig. 1A). This footplate was secured to the motor via an aluminum crank arm with a length, width, and height of 200, 25, and 51 mm, respectively. The ankle's center of rotation was then aligned with the axis of the motor and secured into position at 0.17 rad (10°) of ankle extension. This configuration was selected to be consistent with previous investigations of reflex regulation in the ankle (Gottlieb and Agarwal 1979; Kimura et al. 2003).

### Electromyography

Bipolar, surface electrodes (model #272; Noraxon USA, Scottsdale, AZ) were used to record electromyograms (EMG) in soleus (Sol), medial gastrocnemius (MG), and tibialis anterior (TA). Standard skin preparation techniques were used before applying each electrode to the skin. The resulting signals were amplified using a Bortec® AMT-16 system (Bortec Biomedical, Calgary, AB, Canada), which has a bandwidth of 10–1000 Hz, an input impedance of 10 GΩ, and a common-mode rejection ratio of 115 dB at 60 Hz. Analog signals were antialias filtered using custom-built, differential input, fifth-order Bessel filters with a cutoff frequency of 500 Hz, and then sampled at 2500 Hz with a 16-bit data acquisition system (PCI-DAS1602/16; Measurement Computing Corporation, Norton, MA).

### Maximum voluntary contractions

Maximum voluntary contractions (MVCs) were recorded at the beginning of each experiment; these values were used to normalize EMG activity across subjects. MVCs in ankle extension and flexion were performed against the motor while the ankle was positioned at 0.17 rad (10°) of ankle extension. Two repetitions in each direction were performed for each subject. The MVC for each muscle was computed by taking the maximum average rectified EMG amplitude calculated over a 50-ms moving window.

### Experiment 1: Acceleration dependence of the medium-latency reflex

Ramp and hold perturbations were used to investigate whether stretch reflexes in Sol and MG were sensitive to changes in perturbation acceleration. Some of the details for Experiments 1 & 2 have been described in a previous study from our laboratory (Finley et al. 2012), but are included here for clarity. For experiment 1, perturbation amplitude and peak velocity were fixed at 0.17 rad (10°) and 3.5 rad/sec (200°/sec), respectively, whereas the acceleration was set at either 87 rad/sec^2^, 105 rad/sec^2^, 140 rad/sec^2^, 175 rad/sec^2^, or 873 rad/sec^2^. The amplitude and velocity parameters were selected to be within the range of reported parameters used in prior studies of the stretch reflexes at the ankle (Gottlieb and Agarwal 1979; Grey et al. 2001; Kimura et al. 2003). The maximum acceleration was selected to match data from a prior study where only SLR responses were observed in the ankle extensors (Finley et al. 2012). The remaining accelerations were above the threshold necessary to elicit an MLR and below the acceleration at which MLRs were no longer observed. Across all perturbations, the variation in acceleration resulted in durations of stretch ranging from 50 to 90 msec for the maximum and minimum accelerations, respectively. Although we are unaware of a stretch duration dependence of longer latency responses in the lower limb, each of these durations was greater than the minimum duration necessary to observe LLRs in the upper limbs (Lee and Tatton 1982; Lewis et al. 2005).

Stretch reflexes were elicited under fixed levels of background torque. Before each perturbation, subjects were instructed to generate 5 ± 2% of their MVC in ankle extension using visual feedback from a computer display. The perturbation was triggered only after subjects had maintained the desired torque for a random interval ranging from 1 to 3-sec. There were no limitations on the amount of time subjects had to achieve the desired torque. The data were collected in blocks of 10 perturbations with a 1-min rest period between blocks. Ten perturbations were collected for each acceleration, and the order of accelerations was randomized within each block. Subjects were instructed to not react to the perturbation as it has been demonstrated that longer latency components of the stretch reflexes are sensitive to the instructions provided to the subjects (Hammond 1956; Crago et al. 1976).

### Experiment 2: Task-dependent modulation of the medium-latency reflex

Based on the results from the primary experiment, it was possible to select perturbation characteristics that elicited an MLR in all subjects. The objective of this secondary experiment was to determine if the MLR was enhanced during control of unstable loads. This was accomplished by programming the actuator to act as either a stiff position servo with a stiffness of 35 kNm/rad or an unstable load with properties similar to an inverted pendulum. In the stiff condition, subjects maintained an ankle extension torque of 5 ± 2% of MVC using visual feedback from a computer display. In the unstable condition, subjects maintained an ankle extension angle of 0.170 ± 0.017 rad (10 ± 1°) using visual feedback of ankle angle while controlling a load which behaved like an inverted pendulum centered about the primary axis of the ankle. Details of the visual feedback for both conditions have been described previously (Finley et al. 2012). In the unstable condition, an admittance control algorithm was used to simulate a haptic environment that included an inertial element, an elastic element, and gravity. We chose to simulate a load with nonlinear dynamics to mimic the type of dynamic load that the ankles are responsible for controlling during stance. For all subjects, the mass of the pendulum was set to 25% of the subject's mass and the height was set to 1 m. This mass and height were selected so that subjects could control its position without fatigue. The simulated environment also included an elastic element of stiffness K_env_ in parallel with the pendulum. In addition to these elements, a flexion bias torque equal to 5% of the subject's MVC was applied so that when the subject was in the center of the target, the torque would be matched with the stiff environment. There was no significant difference in the baseline torque between the stiff and unstable environments (stiff: 6.1 ± 0.2%MVC, unstable: 6.6 ± 0.8%MVC, *P* = 0.70). Overall, the relationship between the torque generated by the ankle (τ_ankle_) and the ankle's position was determined from the following equation,


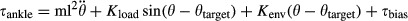


where θ and θ_target_ represent the actual and desired ankle angle and *K*_load_ is equal to -*mgL*. The model parameters were selected to be equivalent to those used in a previous study of SLR regulation during control of unstable loads (Finley et al. 2012). If this relationship is linearized about θ_target_, the relationship between the motor torque and ankle position can be represented as the sum of the load stiffness, *K*_load_, and the stiffness of the environment, *K*_env_. The stiffness of the elastic element (*K*_env_) was equal to 0.5 mgL Nm/rad, where *m* is the mass of the pendulum, *g* is the acceleration due to gravity, and *L* is 1 m. Because *K*_env_ and *K*_load_ have opposite signs, the environmental stiffness counteracts the instability generated by the simulated load.

Stretch reflexes were elicited using ramp and hold perturbations with an amplitude, velocity, and acceleration of 0.17 rad, 2.5 rad/sec, and 175 rad/sec^2^, respectively, values found to consistently elicit an MLR in all subjects participating in the primary experiment. Perturbations were triggered after subjects had maintained the desired torque (stiff condition) or position (unstable condition) for a random interval ranging from 1 to 3 sec. This random interval prevented subjects from systematically activating their muscles before the perturbation. As described in Experiment 1, there were no constraints on the amount of time subjects could take to achieve the target. Across all subjects, 2–10 sec was required to match the torque or position target. We generated identical perturbations by switching the actuator from admittance control to position servo mode at the onset of each perturbation (Shemmell et al. 2009). Each block consisted of a sequence of 20 perturbations and the interval between perturbations was uniformly varied from 1 to 3 sec. We chose to apply ramp and hold stretches because they allowed us to control the kinematic variables known to influence reflexively evoked changes in muscle activation, our primary outcome measure. The results of these studies may be useful for later designing of more complex perturbations for efficiently quantifying the mechanical properties of the ankle (Kearney et al. 1997; Mugge et al. 2010). As described in the first experiment, subjects were instructed to not react following the perturbation.

### Data analysis

Electromyograms were processed by first removing the mean value, then rectifying and normalizing to MVC. The amplitudes of the SLR and MLR were quantified using the averaged, rectified EMG traces for each block. Onset of the SLR was defined as the initial time following perturbation onset when the subsequent EMG exceeded three standard deviations above the average baseline activity for at least 5 msec. The average amplitude of the SLR was computed over a 15-msec window beginning at the onset of the response. We specifically chose a short window to avoid contaminating the SLR with activity from the MLR. As the EMG typically did not return to baseline levels prior to the MLR response, no attempts were made to quantify the actual onset of this response. Instead, we computed the average amplitude of the MLR over a 15-msec window centered about the peak of the MLR response. For each acceleration, the peak latency of the MLR was determined by finding the time point when the amplitude of the average EMG trace was greatest in a window beginning at the end of the SLR window and ending 100 msec after perturbation onset. This latter cutoff was selected to prevent contamination from voluntary responses. For each subject, this peak latency was adjusted for different accelerations to account for any shifts in the timing of the MLR. If the MLR peak occurred less than 8 msec after the SLR window or if no clear peak was observed, the MLR averaging window was set to begin immediately after the window used to compute the SLR.

### Statistical analysis

A linear mixed-effect model, with acceleration as a fixed factor and subject as a random factor, was used to evaluate if there was a significant influence of acceleration (Experiment 1) on reflex latency and amplitude. An analysis of variance (ANOVA) was used to determine significance. Post hoc comparisons between the different accelerations were performed using the Tukey method. For Experiment 2, paired *t*-tests were used to evaluate if there was a significant effect of mechanical environment on reflex amplitude. For each protocol, significance was assessed at the 5% level, and all statistical analyses were performed in the R environment for statistical computing (The R Foundation for Statistical Computing, Vienna, Austria).

## Results

### Experiment 1: Variation in SLR and MLR amplitude with acceleration

**Figure 2:**
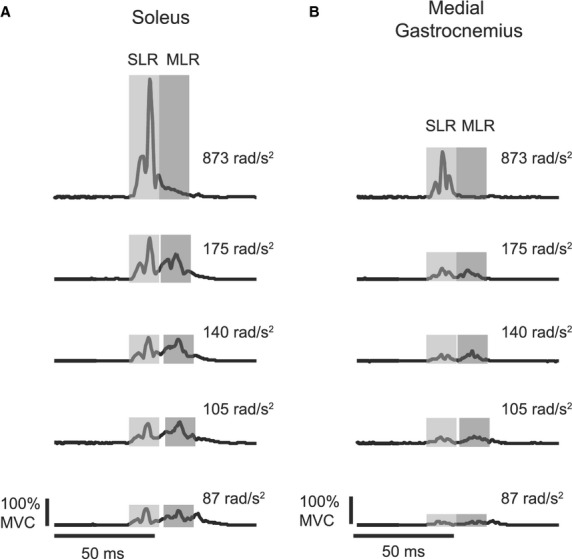
Example from a single subject of the variation in SLR and MLR amplitude for (A) soleus and (B) medial gastrocnemius. Each row represents a different level of acceleration ranging from 87 rad/sec^2^ in the lowest traces to 873 rad/sec^2^ for the top traces. Each trace represents the average of 10 responses and is aligned with perturbation onset. Shaded areas indicate the windows used for computing SLR and MLR amplitude. These windows differed slightly for each acceleration based on timing of the SLR and MLR peaks. Vertical scale bars represent an EMG amplitude of 100% MVC. Horizontal scale bars indicate a duration of 50 msec.

Both SLRs and MLRs were observed following perturbations of varying acceleration (Fig. 2). The earliest Sol response had an average onset and peak latency of 43 ± 3 and 51 ± 3 msec, respectively. For MG, the earliest response began at 40 ± 3 msec and peaked at 47 ± 3 msec. The MLR reached a peak at 71 ± 6 msec for Sol and 68 ± 6 msec for MG. These latencies did not vary across accelerations (SLR: *F*_(1,27)_ = 0.03, *P* = 0.89; MLR: *F*_(1,27)_ = 0.37, *P* = 0.55) and are consistent with those reported previously (Toft et al. 1991).

**Figure 3:**
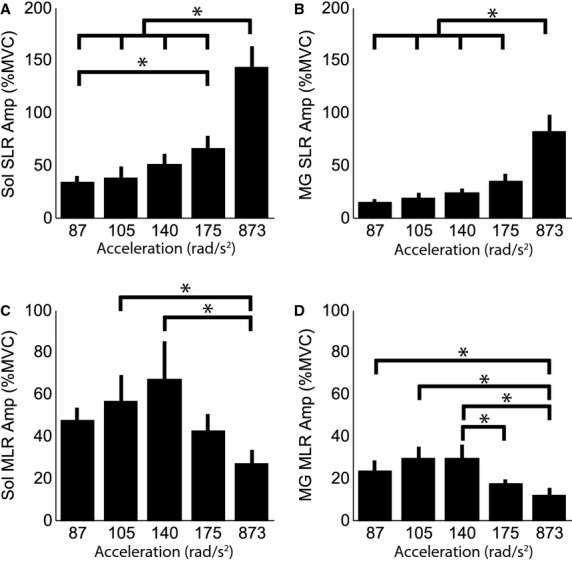
Average SLR and MLR amplitudes for each acceleration. (A) Average SLR amplitude for Sol. (B) Average SLR amplitude for MG. (C) Average MLR amplitude for Sol. (D) Average MLR amplitude for MG. SLR amplitude increased monotonically with acceleration for both Sol and MG. In contrast, MLR amplitude varied in a nonlinear manner as acceleration was increased, peaking at moderate values of acceleration and reaching a minimum at the highest acceleration. Error bars represent standard errors. Asterisks represent significant differences at the 5% level.

Although the SLR is known to be a velocity-dependent response (Powers et al. 1989), we found that SLR amplitude also scaled with acceleration. In the data from our exemplar subject (Fig. 2), the SLR in both Sol and MG was smallest at 87 rad/sec^2^, became larger as acceleration was increased, and reached a peak at 873 rad/sec^2^. This pattern also held for the group (Fig. 3A,B) as SLR amplitude increased monotonically with acceleration. There was a significant influence of perturbation acceleration on the SLR elicited in Sol (*F*_(1,27)_ = 38.46; *P* < 0.001) and MG (*F*_(1,27)_ = 31.35; *P* < 0.001). At an acceleration of 873 rad/sec^2^, the average SLR amplitude was 142 ± 20%MVC and 82 ± 16%MVC for Sol and MG, respectively. In comparison, the amplitude of the SLR for the lowest acceleration (87 rad/sec^2^) was only 34 ± 6%MVC and 15 ± 3%MVC for Sol and MG, respectively. Thus, the amplitude of the SLR at the highest acceleration could be 4–5 times as large as the amplitude measured at the lower acceleration.

Although the SLR amplitude increased with acceleration, the MLR amplitude varied in a nonmonotonic manner. For our representative subject (Fig. 2), the largest MLRs for both Sol and MG were observed in the middle of our acceleration range, from 105 to 175 rad/sec^2^. At the highest acceleration, 873 rad/sec^2^, no distinct MLR was present in either muscle. In Sol, the level of activity during the MLR window was greater than baseline, although it remains unclear if this activity results from an extended SLR or the merging of the SLR and MLR. The nonlinear modulation of the MLR with acceleration was observed in the group data as well (Fig. 3C,D). There was a significant influence of perturbation acceleration on the MLR elicited in Sol (*F*_(1,27)_ = 11.16; *P* < 0.01) and MG (*F*_(1,27)_ = 13.53; *P* < 0.01). For Sol, the MLR grew as the acceleration was increased from 87 to 140 rad/sec^2^, but reduced in amplitude as acceleration reached 873 rad/sec^2^. The amplitude of the Sol MLR at the highest acceleration was significantly less than the amplitude at 140 rad/sec^2^ where the MLR was greatest (140 rad/sec^2^: 67 ± 18%MVC, 873 rad/sec^2^: 27 ± 7%MVC, *P* < 0.001). A similar pattern was observed for MG (140 rad/sec^2^: 29 ± 7%MVC, 873 rad/sec^2^: 12 ± 4%MVC, *P* < 0.001). At 140 rad/sec^2^, when the MLR was largest, its amplitude was comparable to that of the SLR for both Sol (SLR: 50 ± 10% MVC, MLR: 67 ± 18% MVC, *P* = 0.29) and MG (SLR: 24 ± 4%MVC, MLR: 29 ± 7%MVC, *P* = 0.44).

### Experiment 2: Modulation of MLR amplitude during control of unstable loads

**Figure 4:**
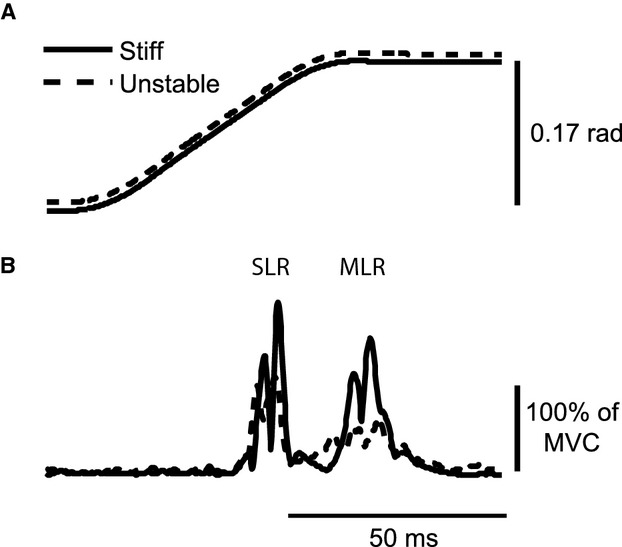
Representative example of SLR and MLR amplitude for Sol during control of an unstable load. (A) Perturbation trajectories for the stiff (solid trace) and unstable (dashed trace) environments. (B) Soleus reflex responses for the stiff (solid trace) and unstable (dashed trace) environments. Each trace represents the average of 20 responses. The amplitudes of both the SLR and MLR were reduced as this subject balanced the unstable load.

**Figure 5:**
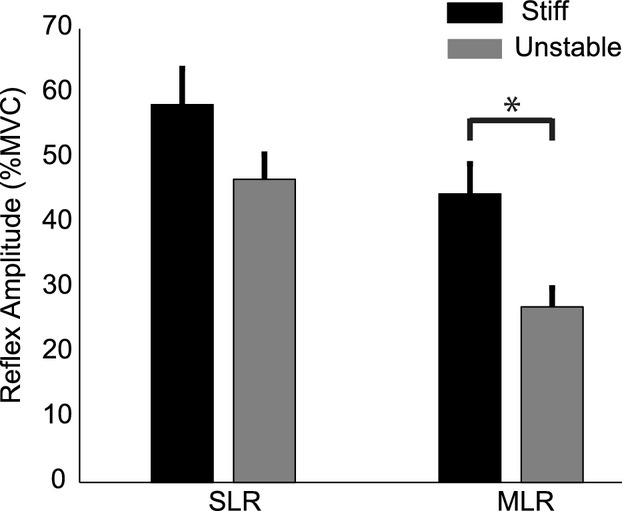
Average SLR and MLR amplitudes during interaction with the stiff and unstable environments. Across all subjects, the MLR was significantly attenuated during control of the unstable load. A similar trend was observed for the SLR, but this difference did not reach statistical significance. Asterisks represent a significant difference at the 5% level.

After determining a set of perturbation parameters that elicited consistent SLRs and MLRs, we found that both the SLR and MLR amplitudes decreased as subjects balanced an unstable load. This is demonstrated by the average Sol reflex responses from a representative subject in both the stiff and unstable environments (Fig. 4). Each response followed a flexion perturbation of 0.17 rad with a peak velocity of 3.5 rad/sec and acceleration of 175 rad/sec^2^. This attenuation of the MLR was consistently observed across subjects (Fig. 5). The average MLR amplitude in the stiff environment was significantly greater than the amplitude in the unstable environment (stiff: 47 ± 4%MVC, unstable: 27 ± 3%MVC, paired *t*-test: *P* < 0.001). A similar trend was observed for the SLR, although it did not reach statistical significance (stiff: 58 ± 6%MVC, unstable: 44 ± 5%MVC, paired *t*-test: *P* = 0.07). The observed changes in MLR amplitude were not due to differences in torque or position prior to the perturbation as these were deliberately matched across the stiff and unstable environments (paired *t*-tests, torque: *P* = 0.70, position: *P* = 0.37). Although torque and position were matched, subjects increased the level of tonic activity in the ankle flexor tibialis anterior (TA) during control of the unstable load (stiff: 0.44 ± 0.07%MVC, unstable: 3.1 ± 1.5%MVC, paired *t*-test: *P* < 0.01). This increase in TA activity was accompanied by increased activity in Sol (stiff: 4.3 ± 0.8%MVC, unstable: 5.8 ± 1.3%MVC, paired *t*-test: *P* < 0.01) and MG (stiff: 2.0 ± 0.4%MVC, unstable: 2.8 ± 0.9%MVC, paired *t*-test: *P* < 0.01) during control of the unstable load. This increase in cocontraction is consistent with previous observations which demonstrated that increased cocontraction could be partially responsible for reduced SLR amplitude during control of unstable loads (Finley et al. 2012). Therefore, it is possible that the attenuation of the MLR may also reflect the inhibitory action of tibialis anterior activation during cocontraction.

## Discussion

The objectives of this study were to identify conditions in which longer latency responses could be elicited in the human lower limb, and to examine how these responses are modulated in tasks that compromise stability. We found that MLR amplitude varied with perturbation acceleration, peaking at moderate accelerations, and then becoming largely attenuated at high accelerations. This acceleration dependence may explain why MLRs are inconsistently observed in studies of the lower limb despite the use of similar perturbation amplitudes and velocities. After finding perturbation parameters that consistently elicited MLRs, we were also able to demonstrate that this response was reduced during control of an unstable load. This finding is in opposition to results reported for the human upper limb, in which LLR amplitude is heightened during tasks that compromise stability (Doemges and Rack 1992b; Dietz et al. 1994; Perreault et al. 2008; Shemmell et al. 2009; Krutky et al. 2010), leading to a corresponding increase in limb stiffness and stability (Doemges and Rack 1992a; Krutky et al. 2013). This suggests that neural strategies for maintaining stability in the lower limbs may be less reliant on rapid, excitatory feedback than the strategies used in the upper limbs.

### Acceleration dependence of the medium-latency stretch reflex

There is substantial inconsistency in the literature regarding the presence of MLRs in the lower limb following perturbations. Some studies report consistent MLRs (Fellows and Thilmann 1989; Toft et al. 1991; Fellows et al. 1993; Grey et al. 2002; Kimura et al. 2003), whereas others report only a single SLR (Allum et al. 1982; Kearney and Chan 1982; Nielsen et al. 1994; Avela et al. 1999; Finley et al. 2012). Given the acceleration dependence demonstrated in this study, it is possible that the perturbation profiles used in previous studies contributed to the variable results that have been reported. Perturbation profiles can be influenced by the bandwidth of the actuator used in each experiment, as well as by the commands sent to the actuator. Our findings suggest that it is important to report the measured acceleration, in addition to the commonly reported displacement and velocity, to facilitate comparisons across studies examining the reflex responses to externally imposed perturbations. This idea has also been suggested for studies of standing posture as postural responses to surface translations also vary with acceleration (Welch and Ting 2009).

Our conclusions regarding the relationship between MLR amplitude and acceleration are similar to those of Berardelli et al. (1982), although significant differences exist between the two studies. The most important difference is that in this study, we controlled perturbation acceleration independently from peak velocity. Although acceleration was quantified in the former study, their observations could potentially be explained by concurrent changes in velocity. Here, we have demonstrated that the variation in MLR amplitude was a result of changes in acceleration as the joint experienced the same peak velocity in all conditions. Another key difference between this study and past work is that the nonlinear association between reflex amplitude and acceleration is ascribed to responses that occurred in two different time windows. In the study by Berardelli et al. (1982), the response that varied nonlinearly with acceleration occurred at a minimum latency of 93 msec and was only present in 30-40% of the subjects tested. The initial longer latency response they observed, which is comparable in latency to MLR in this study, increased monotonically with acceleration. This difference is likely due to the limited acceleration range used in their study. Their peak acceleration, ∼ 80 rad/sec^2^, was within our lower acceleration range where we also observed a monotonic increase in MLR amplitude. As we used perturbations that covered a higher range of accelerations, we were able to demonstrate the nonlinear variation in LLR with changes in acceleration.

### Possible neural mechanisms responsible for acceleration dependence of stretch reflexes

It is possible that the properties of peripheral receptors underlie the acceleration dependence of the SLR and MLR. Although the Ia fibers responsible for the SLR are highly sensitive to stretch velocity (Matthews 1964), these fibers exhibit an early peak in activity at the onset of stretch corresponding to the time when acceleration would be greatest (Houk et al. 1981; Haftel et al. 2004). Moreover, the amplitude of this transient scales with acceleration magnitude (Schafer 1967). A similar acceleration-dependent scaling of Ia afferent activity has been observed in humans during dynamic grasping and key-pressing tasks (Dimitriou and Edin 2008a,b). Therefore, it is possible that this activity is responsible for the acceleration-dependent scaling of the SLR observed in this study.

This acceleration dependence of Ia afferent activity may explain the attenuation of the MLR at high accelerations. As acceleration increases, a larger population of motoneurons would be recruited leading to an increase in SLR amplitude. As recruitment of these motoneurons is likely to be synchronized, this would result in a subsequent synchronization of their refractory periods, potentially leaving them unresponsive during the time when an MLR would typically be observed. At lower accelerations, a smaller population of motoneurons would be recruited, leaving a portion of the motoneuron pool available to fire in response to subsequent afferent volleys during the MLR time window. In line with this explanation, a recent study has provided evidence that motoneuron synchronization may underlie the stretch duration effect on the LLR in the biceps (Schuurmans et al. 2009).

Force-sensitive afferents are another possible source for the acceleration-dependent behavior of the MLR. Ib afferents from Golgi tendon organs respond to forces generated by muscle contraction (Houk and Henneman 1967) and typically inhibit homonymous and synergistic muscles (Eccles et al. 1957; Pierrot-Deseilligny et al. 1979; Nichols 1989). As our subjects maintained a tonic contraction at perturbation onset, it is possible that the large initial accelerations we used produced a rapid increase in muscle force and subsequent inhibition of the ankle extensors. There is also evidence of positive force feedback from load-sensitive receptors during stance (Dietz 1992; Pratt 1995),

Stretch duration is another factor that could potentially contribute to the acceleration dependence of MLR amplitude. Lee and Tatton (1982) demonstrated that the LLR at the wrist joint was only observed following perturbations with a duration greater than a critical time of about 40 msec. This duration dependence was also reported for the more proximal biceps which has a critical duration of 36 ± 5 msec (Lewis et al. 2005). Our perturbation durations ranged from 50 to 90 msec. If the critical duration for the ankle extensors is longer than what has been established for the elbow and wrist, it is possible that our perturbations with the highest acceleration (shortest duration) were not of sufficient duration to elicit an MLR. However, given that the perturbations with the lowest acceleration (longest duration) also failed to elicit MLRs, we do not think that duration-dependent threshold effects fully explain the variation in MLR amplitude with acceleration in our study.

### Factors contributing to the reduction in MLR amplitude during control of unstable loads

In contrast to studies of LLR behavior in the upper limb (Doemges and Rack 1992a,b; Dietz et al. 1994; Perreault et al. 2008; Shemmell et al. 2009; Krutky et al. 2010), we found that MLR amplitude in the lower limb was attenuated during a task that required active stabilization. In a prior study (Finley et al. 2012), we demonstrated that the reduction in SLR amplitude during control of unstable loads could be attributed to inhibition resulting from cocontraction of the ankle flexor, tibialis anterior, along with the ankle extensors. This reduction in reflex amplitude may have stemmed from presynaptic inhibition of Ia terminals from descending sources (Nielsen and Kagamihara 1993; Nielsen et al. 1995). Although cocontraction was also observed in this study, it is not clear if the MLR was mediated by Ia afferents. In fact, there is considerable evidence that the MLR in the ankle extensors is mediated by Group-II spindle afferents (Corna et al. 1995; Schieppati and Nardone 1997; Grey et al. 2001). Although it is possible that presynaptic mechanisms may inhibit transmission from Group-II afferents, our study was not designed to identify if the attenuation of MLR was mediated by the same mechanism as the observed SLR attenuation. Irrespective of the precise mechanism, the observed reduction in MLR amplitude during control of unstable loads is consistent with our previous suggestion that reflex contributions to ankle stability may be reduced in situations where stability is compromised.

One issue that remains to be addressed is whether known transcortical reflex pathways contributing to the activation of lower limb muscles exhibit the same stability-dependent modulation as do such pathways involved in the control of upper limb muscles. A number of studies have demonstrated that the LLR in the upper limb is mediated in part by a transcortical loop (Evarts 1973; Cheney and Fetz 1984; Shemmell et al. 2009; Pruszynski et al. 2011). In contrast, it has been suggested that the MLR in the ankle extensors is mediated by segmental transmission from Group-II afferents (Dietz 1992; Corna et al. 1995; Nardone et al. 1996; Schieppati and Nardone 1999; Grey et al. 2001). If facilitation of the LLR during tasks that challenge stability is a fundamental property of transcortical feedback loops, then facilitation should also be in observed lower limb feedback loops that incorporate the cortex. Tibialis anterior exhibits a late burst of activity (after 90–100 msec) in response to stretch, known as an LLR, and this response is believed to involve a transcortical loop (Petersen et al. 1998; Christensen et al. 2001; Van Doornik et al. 2004). A similar third response can occasionally be observed in the ankle extensors (Gottlieb and Agarwal 1980; Gottlieb et al. 1983; Sinkjaer et al. 1996) and there is evidence that this response may also be mediated by the cortex (Sinkjaer et al. 1999; Taube et al. 2006). Thus, assessing the behavior of these later responses during control of unstable loads may help to address the discrepancy between the results presented here and those from previous studies in the upper limb.

## Conclusion

Our results provide further support for the idea that the nervous system reduces its reliance on rapid feedback during challenging postural tasks involving the lower limbs. Reduced reflex amplitude during unstable tasks has now been demonstrated via the H-reflex (Llewellyn et al. 1990; Solopova et al. 2003; Taube et al. 2008), the SLR (Finley et al. 2012), and the MLR in the ankle extensors. In order to successfully perform these tasks, the nervous system likely engages higher centers to regulate limb mechanics and stabilize posture. Indeed, responses to postural perturbations at latencies closer to voluntary reaction times (∼120 msec) may be more responsible for stabilizing posture (Jones and Watt 1971; Nashner 1976).
